# An ultrasonic technology for production of antibacterial nanomaterials and their coating on textiles

**DOI:** 10.3762/bjnano.5.62

**Published:** 2014-04-28

**Authors:** Anna V Abramova, Vladimir O Abramov, Aharon Gedanken, Ilana Perelshtein, Vadim M Bayazitov

**Affiliations:** 1 Institute of general and inorganic chemistry of the Russian Academy of Sciences, Leninskiy prospect 31, Moscow, 119991, Russian Federation; 2 Department of Chemistry, Bar Ilan University, Ramat-Gan, 52900, Israel

**Keywords:** antibacterial textile, cavitation, electrical discharge in liquid, nanoparticle, ultrasound

## Abstract

A method for the production of antibacterial ZnO nanoparticles has been developed. The technique combines passing an electric current with simultaneous application of ultrasonic waves. By using high-power ultrasound a cavitation zone is created between two zinc electrodes. This leads to the possibility to create a spatial electrical discharge in water. Creation of such discharge leads to the depletion of the electrodes and the formation of ZnO nanoparticles, which demonstrate antibacterial properties. At the end of this reaction the suspension of ZnO nanoparticles is transported to a specially developed ultrasonic reactor, in which the nanoparticles are deposited on the textile. The nanoparticles are embedded into the fibres by the cavitation jets, which are formed by asymmetrically collapsing bubbles in the presence of a solid surface and are directed towards the surface of textile at very high velocities. Fabrics coated with ZnO nanoparticles by using the developed method showed good antibacterial activity against *E. coli*.

## Introduction

Currently, the problem of nosocomial (acquired in hospitals) infections becomes more and more urgent. About 5–10% of all patients in hospitals are affected by them. Hospital-acquired infections are one of the ten most frequent causes of death. The economic loss caused by nosocomial infections is significant. In the Russian Federation it may reach 10–15 billion RUB per year (conservative estimation). For comparison, the annual economic impact of nosocomial infections in Europe is around 7 billion EUR and in the US about 6.5 billion USD. Hospital-acquired infections significantly reduce the life quality of the patients of life and lead to a loss of reputation for the hospital [1]. In order to reduce the hospital acquired infections, the staff sterilizes surfaces, employs hygiene measures and minimizes contacts between patients. However, reusable textiles such as the patients linen or the doctor robes remain a significant source of infection. To ameliorate the problem one could replace reusable textiles with disposable items. But this is quite expensive. Another more promising approach is to use antibacterial textiles. In this case it is very important to ensure the preservation of the antibacterial properties after washing. Antimicrobial textiles can be produced by coating textiles with antibacterial nanoparticles (NPs). NPs such as zinc oxide NPs are known to have antibacterial properties due to OH**^•^** radicals, which result from defects in their crystal structure [2].

In the case of power ultrasound, cavitation bubbles emerge when the cavitation threshold is exceeded. A sonochemical reaction happening upon the collapse of the acoustic bubble yields a product having nanometric size. The rapidly moving surface of the cavitation bubble, the dynamics of which determines the main characteristics of the coating process, accelerates the nanoparticles. Due to the asymmetric collapse of the bubble and the formation of liquid jets in the presence of a solid surface, the formed jets push the nanoparticles towards the surface at very high velocities (larger than 500 m/s). Scientists at Bar Ilan University [3] demonstrated this deposition technique for the first time by coating submicron silica spheres with Ni nanoparticles. Using this technique antibacterial ZnO or other metallic oxide NPs can be embedded into the textile. The nanoparticles can be produced in a sonochemical reaction described elsewhere [2–3], but on an industrial scale this method requires large amounts of ethanol, whose vapours are dangerous to people and the environment. Thus the goal of the current research is to produce an aqueous suspension of ZnO NPs directly before their introduction into the fibres, deposit them on textile samples and analyse the antibacterial properties of the samples comparing their antibacterial activity and the antibacterial activity of samples coated with industrially produced NPs.

In the research described in this paper we produced a suspension of zinc oxide NPs in water by using a sonoplasma discharge between two Zn electrodes in water. Preliminary experiments have shown that if ultrasonic vibrations are applied to an electrode while an arc discharge is created in polar fluids a new form of an electrical discharge, a spatial sonoplasma discharge, is formed [4–5]. It is a form of a quasi-spatial discharge in liquid, formed in the gap between the electrodes where the ultrasonic vibrations lead to the formation of cavitation bubbles. If certain parameters of the electrical circuit and certain intensity of the ultrasonic field are achieved, the plasma discharge can be formed in the whole volume of the bubbly liquid between the electrodes. The experiments also revealed that the sonoplasma discharge is characterized by a glow in the whole volume of the liquid and an increasing current–voltage characteristic, which is typical for the abnormal glow discharge. Arc discharges in aqueous electrolytes, which are widely used in the industry, are the best known type of a stationary plasma discharge in liquids [6]. Currently this discharge is applied in physical and chemical studies and in the synthesis of different materials, but due to small effective volume of the discharge zone the rate of the processes is quite low. The use a spatial sonoplasma discharge can accelerate the speed of this process.

The current paper reports on the experimental production of ZnO NPs by oxidation of the Zn electrodes induced by the sonoplasma discharge. In continuation of this research a sonication machine for the treatment of textiles in a roll-to-roll mode [7] of operation was used to coat cotton fabrics with ZnO NPs. The testing results of the antibacterial activity of the coated fabric samples are reported and compared with the antibacterial activity of the fabric coated by using the same ultrasonic method [2,7–9] with industrially produced ZnO NPs.

## Experimental

**Figure 1:**
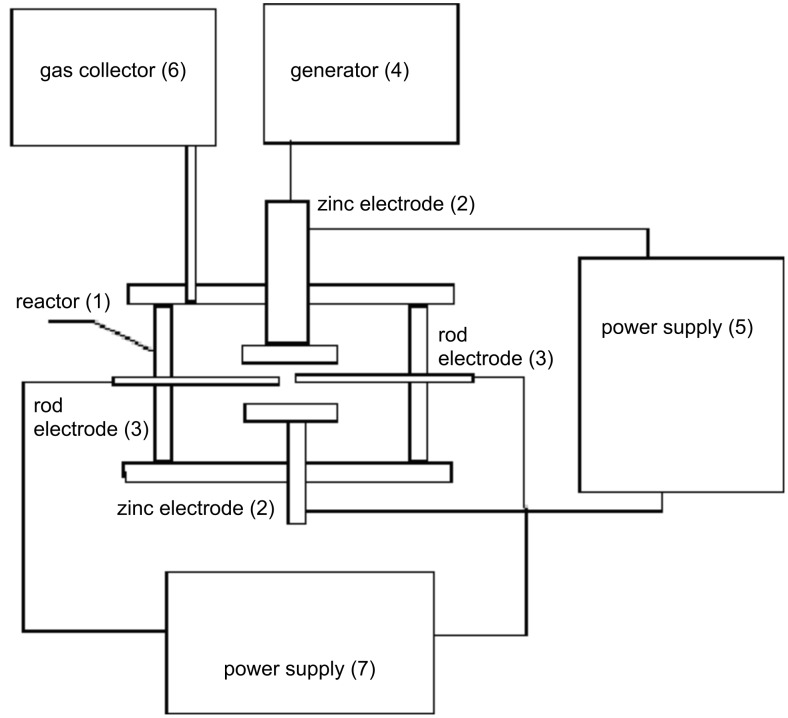
Block diagram of the experimental setup for the production of ZnO NPs in a sonoplasma discharge.

We have built the experimental setup [10] shown in Figure 1 to study the possibility of producing ZnO NPs in a sonoplasma discharge in water. The setup contained a reactor (1) with a working volume of 1 LS. Zinc electrodes (2) were introduced into the reactor. The upper electrode was simultaneously an emitter of the oscillation system, which contained also a waveguide and an ultrasonic transducer. An ultrasonic generator (4) powered the transducer. The electrodes were connected to the power supply of the sonoplasma discharge (5). The setup also contained rod electrodes (3) to initiate the arc discharge, which were connected to their own power supply (7). Gaseous reaction products were accumulated in a special gas collector (6).

The output of acoustical power of the system was 2.0 kW, the working frequency of the transducer and waveguide was 18 kHz. The parameters of acoustical equipment allowed for reaching an intensity of ultrasonic radiation of 10 W/cm^2^ in the liquid. We have used a capacitor C_1_ as the power supply of the sonoplasma discharge. The discharge was initiated by a high voltage pulse in the secondary winding of the transformer TH1. The pulse was initiated by a controlled discharge of the capacitor C_2_, which was connected to the primary winding of the transformer. The capacitor C_2_ was charged by a voltage of 5–10 kV, the transformer ratio of TH1 was 4:1. We have used a heat exchanger to maintain the operating temperature of 60 °C. The experimental production of ZnO NPs was done as follows: We switched on the ultrasonic generator to create a cavitation zone between the electrodes. Then we switched on the discharger that commutes capacitor C_2_ to the primary winding of the pulse transformer. The voltage pulse occurring on the secondary winding induced an electrical discharge in the gap between the electrodes in the reactor. The induced discharge was maintained by the capacitor C_1_ charged to a voltage of 400 V. During such work of the setup we produced a suspension of ZnO NPs in the reactor.

We have studied the morphology of the structure, the chemical and phase composition of the settled NPs using an electron microscope (CAM SCAN S2) and an X-ray spectral microanalyzer. Studies of the particle size distribution were carried out by using DLS measurements. X-ray diffraction was performed on the diffractometer “AMUR-K”. The instrument was equipped with a one-coordinate position-sensitive detector OD2 for the fixed wavelength λ equal to 0.1542 nm and a Kratky collimation system. The cross-section of the X-ray beam was 0.2 × 8 mm, the area of scattering angles corresponded to the scattering-vector range of 0.1 < *s* < 5.0 nm^−1^ (*s* = (4π sin θ)/λ, with 2θ being the scattering angle). Samples of the particles were placed in a test cell of polyethylene terephthalate film with a thickness of 20 μm. The measurement procedure was performed using a certified methodology approved for the machine "AMUR-K" [11].

We have used the reactor described in [7] to produce antimicrobial textiles coated with nanoparticles. Ultrasonic vibrations were introduced into the reactor through two magnetostrictive transducers with an operating frequency of 19 kHz, one of which was located above the moving fabric, and the other one below it. Magnetostrictive transducers were welded onto rectangular steel plates to increase the surface area for irradiation. The vibrations of the plates were transmitted directly into the reaction zone. The power of each transducer was 2.5 kW, which was enough to initiate cavitation in the reactor. The speed of the textile (which was 100% cotton) was 1.5 m/min. We have produced two sets of coated fabrics. The first set was coated with industrial NPs (ZnO NPs dispersion in H_2_O, Sigma Aldrich). An aqueous suspension with the concentration of 1.125 g ZnO per litre of distilled water was prepared. In the second set the suspension produced by the sonoplasma discharge was immediately used to impregnate the textile. The same concentration of ZnO NPs was used in both experiments. The antibacterial activity of the two sample sets against *E. coli* was tested by using the standard method BS EN ISO 20743:2007 [12]. We have calculated the antibacterial activity according to the following formula:


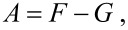


where *F* is the growth rate of bacteria in the control sample (log_10_ CFU/mL after incubation − log_10_ CFU/mL before incubation), *G* is the growth rate of bacteria on the coated samples.

## Results and Discussion

**Figure 2:**
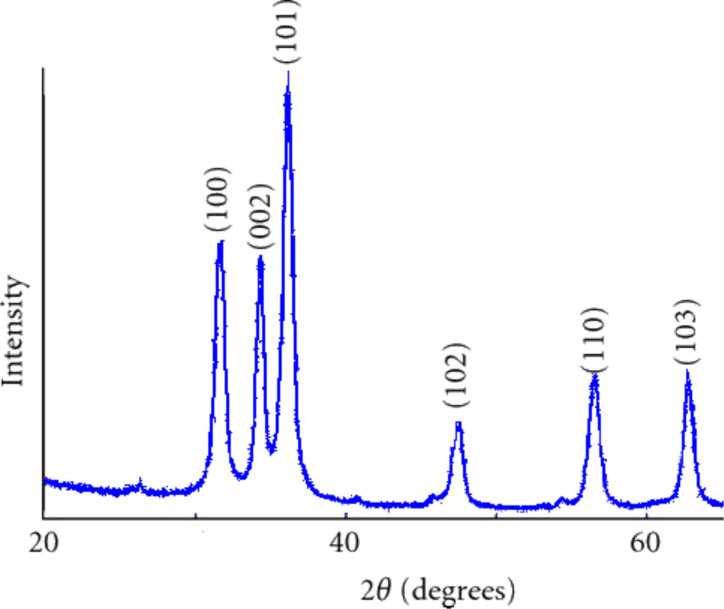
XRD pattern of the produced particles. The reflections were indexed according to the diffraction pattern of hexagonal wurtzite-type ZnO.

In order to produce a suspension of ZnO NPs in water we have initiated a sonoplasma discharge with the described above parameters in the experimental setup. An X-ray diffraction analysis of the obtained suspension confirmed that ZnO NPs were produced. Figure 2 shows the X-ray diffraction pattern. The reflections were indexed according to the diffraction pattern of hexagonal wurtzite-type ZnO.

**Figure 3:**
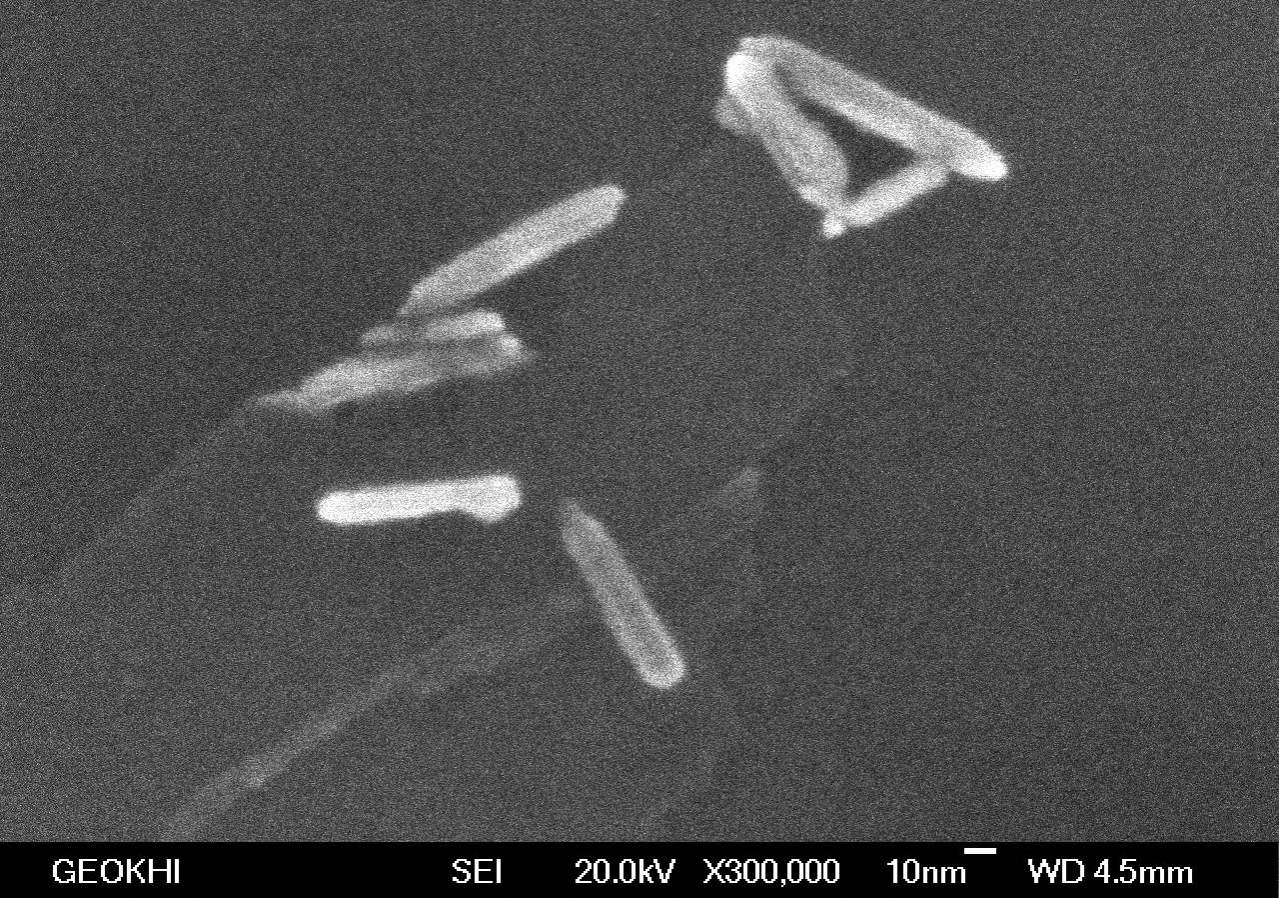
SEM image of ZnO NPs produced in a sonoplasma discharge. The scale bar is 10 nm.

We have investigated the morphology and particle size by electron microscopy. Figure 3 shows the SEM image of the product. The SEM image shows that the produced particles have a cylindrical shape and confirms that their radius is about 10 nm. The length of these rods is about 50 nm.

**Figure 4:**
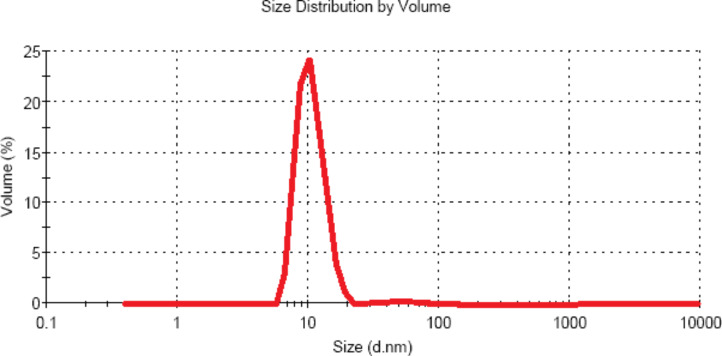
Size distribution of the zinc oxide particles produced in the sonoplasma discharge.

We have obtained the size distribution of the zinc oxide particles produced in the sonoplasma discharge by using DLS measurements (Figure 4). The results confirm that a stable monodispersed suspension of ZnO NPs can be produced by using a sonoplasma discharge. A narrow size distribution of particles with an average size of 10 nm is obtained from the DLS measurements, which is in good agreement with the SEM data.

**Figure 5:**
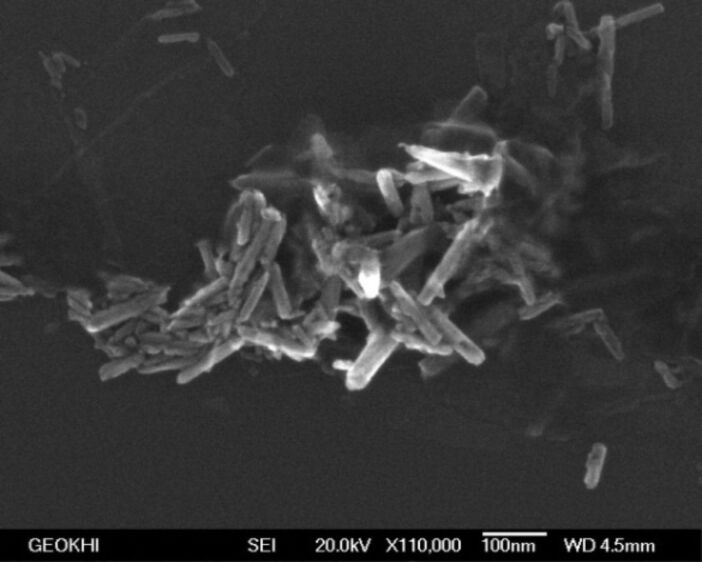
A SEM image of the agglomerates of ZnO NPs in the suspension 1 h after the discharge. The scale bar is 100 nm.

It was relevant for the coating process that the suspension produced in the sonoplasma discharge was used for coating immediately after production. Otherwise the nanoparticles formed agglomerates with the average size of 1–5 μm. This fact was confirmed by using SEM imaging. Figure 5 shows a SEM image of the agglomerates that appeared in the suspension after 1 h.

**Figure 6:**
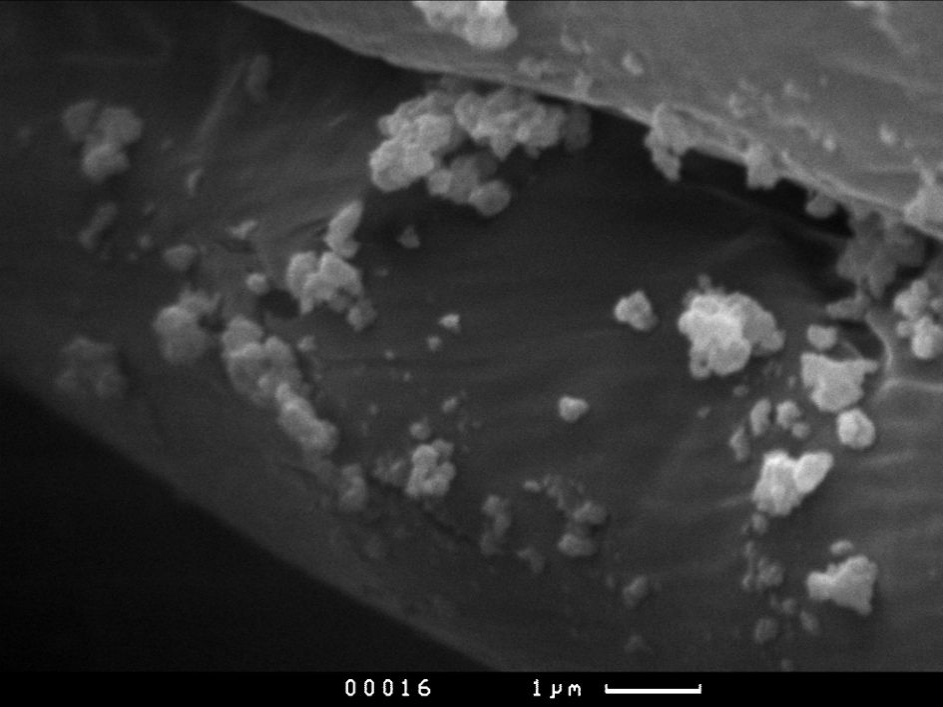
A SEM image of the coated textile fibres. The scale bar is 1 μm.

Two sets of fabric, one coated with industrial nanoparticles and the second one with nanoparticles fabricated in the sonoplasma discharge, were produced. Figure 6 shows a SEM image of the coated fabric. The sample shown was coated with commercial nanoparticles. It is clearly visible that the commercial nanoparticles can reach up to 1000 nm.

**Figure 7:**
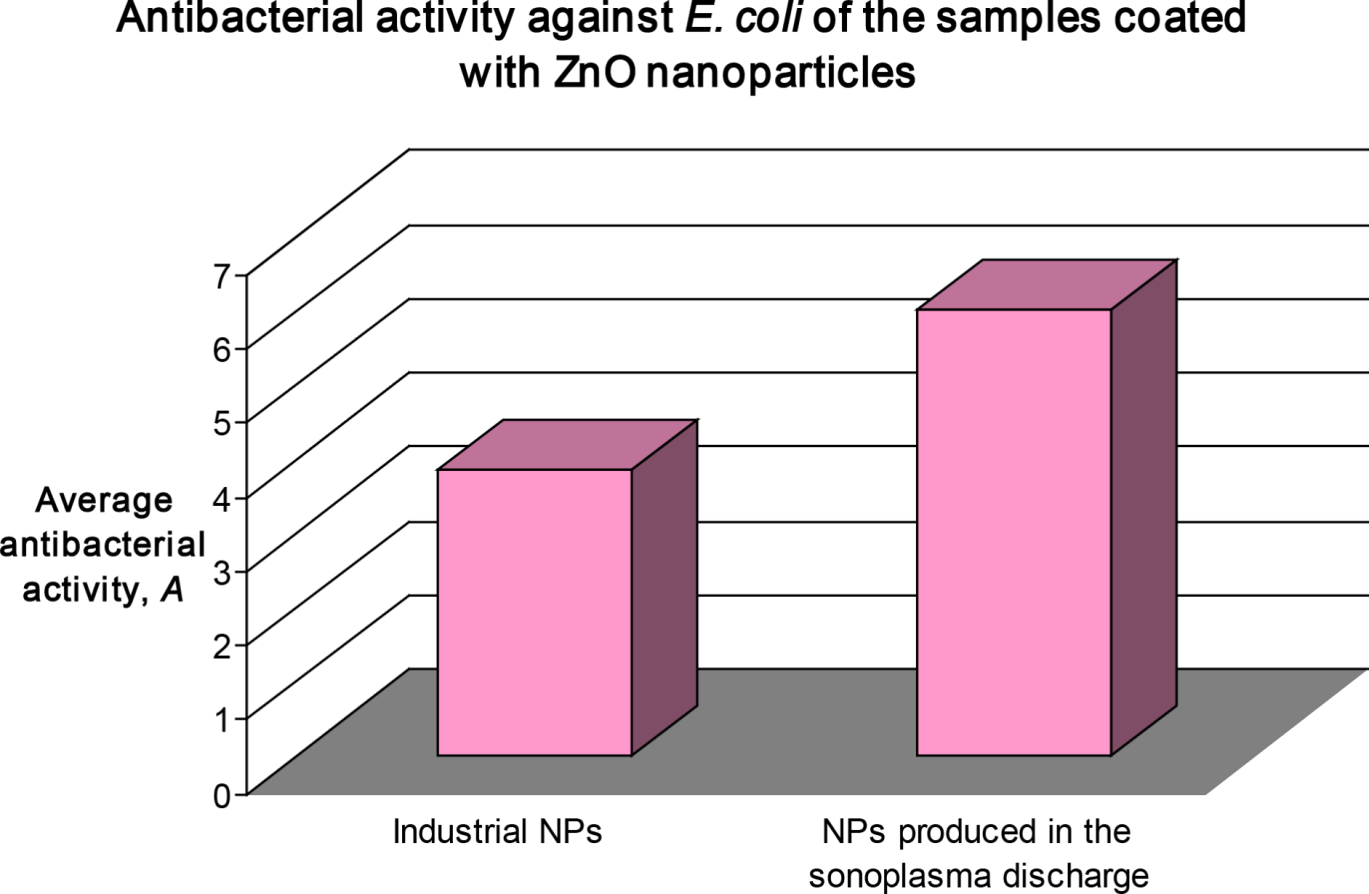
Antibacterial activity against *E. coli* of the samples coated with ZnO nanoparticles.

We have tested the antibacterial activity against *E. coli* of the two sets of fabrics. Figure 7 shows the results of these tests. It is clearly visible that the antibacterial activity of the textile coated by the sonoplasma particles against *E. coli* is higher than the fabric coated with industrial NP’s. This might be explained as the result of the small ZnO NPS obtained by the sonoplasma synthesis. We have repeatedly shown that the biocidal effect is size dependent and particles with smaller size kill bacteria better. It is also advantageous that the suspension is continuously exposed to an ultrasonic field, which prevents the particles from forming agglomerates.

## Conclusion

A method for the production of antibacterial ZnO nanoparticles has been developed. By using high power ultrasound a sonoplasma discharge is created between two zinc electrodes. Cylindrical rod-shaped ZnO nanoparticles with dimensions of 10–50 nm are formed in such a discharge. If the suspension of nanoparticles obtained in the sonoplasma reactor is immediately transported to a special reactor for coating of textiles with nanoparticles, antibacterial textiles can be produced. This coated textiles show better antibacterial activity against *E. coli* than textiles that were coated in the same reactor with industrially produced nanoparticles.
